# Can favourable psychosocial working conditions in midlife moderate the risk of work exit for chronically ill workers? A 20-year follow-up of the Whitehall II study

**DOI:** 10.1136/oemed-2017-104452

**Published:** 2017-10-17

**Authors:** Maria Fleischmann, Ewan Carr, Stephen A Stansfeld, Baowen Xue, Jenny Head

**Affiliations:** 1 Department of Epidemiology and Public Health, University College London, London, UK; 2 Institute of Psychiatry, King’s College London, London, UK; 3 Centre for Psychiatry, Wolfson Institute of Preventive Medicine, Barts and the London School of Medicine and Dentistry, Queen Mary University of London, London, UK

**Keywords:** chronic disease, psychosocial working conditions, retirement, older workers, work exit

## Abstract

**Objectives:**

To investigate if favourable psychosocial working conditions can reduce the risk of work exit and specifically for workers with chronic disease.

**Methods:**

Men and women (32%) aged 35–55, working and having no chronic disease at baseline of the Whitehall II study of London-based civil servants were selected (n=9040). We observed participants’ exit from work through retirement, health-related exit and unemployment, new diagnosis of chronic disease (ie, coronary heart disease, diabetes, stroke and cancer) and their psychosocial working conditions in midlife. Using cause-specific Cox models, we examined the association of chronic disease and favourable psychosocial working conditions and their interaction, with the three types of work exit. We adjusted for gender, occupational grade, educational level, remaining in civil service, spouse’s employment status and mental health.

**Results:**

Chronic disease significantly increased the risk of any type of work exit (HR 1.27) and specifically the risk of health-related exit (HR 2.42). High skill discretion in midlife reduced the risk of any type of work exit (HR 0.90), retirement (HR 0.91) and health-related exit (HR 0.68). High work social support in midlife decreased the risk of health-related exit (HR 0.79) and unemployment (HR 0.71). Favourable psychosocial working conditions in midlife did not attenuate the association between chronic disease and work exit significantly.

**Conclusions:**

The chronically ill have increased risks of work exit, especially through health-related exit routes. Chronic disease is an obstacle to extended working lives. Favourable working conditions directly relate to reduced risks of work exit.

**What this paper adds**

- Older workers with chronic disease generally exit work earlier than healthy workers.
- Few studies have investigated if favourable psychosocial working conditions can reduce the risk of workers with chronic disease to leave work.
- Workers with chronic disease had increased risks of work exit, but favourable psychosocial working conditions in midlife did not attenuate this risk.
- High skill discretion as well as high work social support in midlife directly reduced the risk of work exit.

## Introduction

To account for the increasing pension spending and to sustain the welfare state, many European countries have in the last decade augmented state pension age and took other measures to discourage early retirement. These changes imply that older workers, including those with chronic disease, may have to work for longer.

Chronic diseases (eg, diabetes, cardiovascular diseases) are prevalent among older persons. In the UK, about 46% of the population aged 55–64 years had a long-standing illness or health problem in 2014.^1^ The OECD report that the employment rate among people with chronic disease is considerably lower compared with people without chronic disease. Among persons aged 50–59 years in OECD countries, the average employment rate for those without chronic disease was 74%, whereas this dropped to around 70% and 50% for people reporting one chronic disease and two or more chronic diseases, respectively.^2^ Many studies show that poor health is related to early exit from work,^3–5^ especially exit due to disability^6–10^ and unemployment or inactivity.^11^ Persons with chronic disease were more likely to leave work early^12 13^ while not being diagnosed with chronic disease contributed to working beyond pensionable age.^14^

In a societal context where working lives are extended, it is important to acknowledge that requirements of older workers with chronic disease may be different compared with workers in good health,^13^ for example because their work capability might be reduced.^15^ In order to accommodate their changing work needs and to provide the basis for extended working lives, adaptions to psychosocial working conditions should be considered. The well-known job demands-control model^16^ implies that modifying individuals’ psychosocial working conditions provides one possibility to reduce individual job strain and increase organisational output. Several authors have used this model in the context of retirement to explain how more unfavourable working conditions push individuals towards retirement^17^ and how promoting working conditions might encourage workers to stay in work.^18^

More favourable working conditions might reduce the risk of work exit and alleviate the burden of work for the increasing number of older workers with chronic disease. In this paper, we aim to investigate (1) whether favourable psychosocial working conditions reduce the risk of work exit generally and (2) whether, for those with chronic disease, favourable working conditions can attenuate their elevated risk of work exit. We hypothesise that more favourable working conditions reduce the risk to exit work and that they moderate the negative relation between chronic disease and work exit.

This research contributes to the existing literature in several ways. First, it provides additional insights into the association of psychosocial working conditions and work exit. Prior publications on this topic are increasing, but report rather inconsistent findings. For example, some studies report that social support was related to later retirement intentions and behaviour,^19^ retirement intentions only,^20^ whereas others do not find significant associations with retirement intentions or behaviour.^6 20 21^ Higher job demands (psychosocial demands or job pressure; not physical demands) were related to earlier intended retirement, but not to retirement behaviour.^19 20^ Some authors reported that high psychosocial job demands related to disability retirement^7^ but others did not find a similar association.^8 22^ Similar inconsistencies between studies and findings regarding intended and actual retirement can be reported for other psychosocial working conditions, such as job rewards and job resources.^20 23–25^

Second, this paper investigates the potential moderating effect of psychosocial working conditions for the relation between chronic disease and work exit. We use validated measures of four chronic diseases, that is, coronary heart disease (CHD), stroke, cancer and diabetes. To our knowledge, only three studies—all conducted in the Netherlands—report that favourable working conditions might attenuate the negative relation between chronic diseases and work exit or sickness absence.^13 22 26^ Among workers with chronic disease, these authors find higher autonomy to be associated with reduced risk of sickness absence^26^ and disability retirement.^22^ Moreover, for this group low psychosocial resources at work (ie, low autonomy, low supervisor or coworker support) are related with higher risk of work exit.^13^ However, other working conditions, such as psychosocial work demands or social support, did not act as significant moderators.^13 22^ In addition to these prior studies, we measure favourable working conditions in midlife, allowing us to assess long-term effects of psychosocial working conditions.

Finally, we investigate the relevance of being diagnosed with chronic disease for work exit in a prospective panel study. Our follow-up period covers approximately 20 years and participants are observed about 14 years on average before leaving work. This comprehensive information allows us to study the onset of chronic disease for work exit, rather than comparing participants without and with chronic disease.

## Methods

We used data from phases 1–9 of the Whitehall II cohort study, established in 1985–1988 (phase 1) and described in detail elsewhere.^27 28^ The sample at phase 1 included 10 308 London-based, male and female civil servants, aged 35–55. Two-thirds of the respondents were men. Follow-up interviews were conducted every 2–3 years. Response rates varied between 71% and 87%. Data collections alternated between postal questionnaires alone (phases 2, 4, 6 and 8) and combined postal questionnaires and clinical examination (phases 3, 5, 7 and 9).

### Sample selection

From the baseline (phase 1) sample of 10 308, we excluded those who exclusively participated in the first phase of Whitehall II (n=786) or left work before phase 3 (n=206). Moreover, we excluded participants who already had a chronic condition at baseline (n=276). Our analytical sample refers to 9040 unique respondents (45 926 repeated observations).

### Route of work exit

In phases 3–9, respondents’ employment status and route of work exit was determined by self-report. Participants who reported that they were working in the civil service or working outside the civil service were coded as being in work. Participants who were not working during follow-up self-reported the reason for not working as ‘unemployed’, ‘retired’, ‘long-term sick’ or ‘other’ (at phase 5, the ‘other’ category is subdivided into doing housework, being student, other; at phases 7 and 9, the ‘other’ category differentiates looking after family/home responsibilities, other). Additionally, those who were ‘retired’ could indicate whether this was ‘retirement on health grounds’. Based on this information, we derive four exit routes from paid work: ‘retirement’ (reported being retired, not on health grounds), ‘health-related exit’ (reported being long-term sick or retirement on health grounds), ‘unemployment’ (reported being unemployed) and ‘other exit’ (other reasons for work exit). Participants who left but later returned to work are coded as working. For participants with multiple exits from work (about 10%), the route of work exit refers to their last exit from paid work.

### Age of work exit

Participants who had left the civil service between two phases reported in which year this was (for respondents who had left the civil service through retirement, this refers to their year of retirement). Based on their year of birth, we calculated their exit age (possible for 66% of the cases). If participants’ exact exit year was unknown, we used the mid-point between their age in the last available phase still in work and the first phase out of work. Participants who were still working at the end of follow-up or left the study (non-participation or death) before leaving work were treated as right-censored. The earliest reason for right-censoring was used.

### Chronic disease

We defined having chronic disease as either one or several of the following conditions: diabetes, CHD (excluding self-report), all stroke and all malignant cancers. All chronic diseases are validated measures. Participants with diabetes were diagnosed using an oral glucose tolerance test in one of the clinical examinations between phases 3 and 9 (WHO definition),^29^ self-reported doctor-diagnosed diabetes or use of antidiabetic medication.^30^

CHD included diagnoses for fatal CHD (International Classification of Diseases revision 9 (ICD 9) codes 410–414 or revision 10 (ICD 10) codes I20-I25; excluded from the study), non-fatal myocardial infarction and ‘definite’ angina. Information on non-fatal myocardial infarction and angina was obtained from several sources. From 1989, the National Health Service (NHS) Hospital Episode Statistics database has provided reports of participants’ diagnoses on discharge and procedure codes for all NHS hospitals in England and Wales. Participants also self-reported CHD events in our health survey questionnaires. These were then validated using the study resting ECGs, the Hospital Episode Statistics database and by contacting general practitioners for confirmation when no other external source exists. We only included validated self-reports of CHD events in our measure of CHD. ‘Definite’ angina included self-reported cases of angina only if they were subsequently validated by these other sources.

Stroke was assessed by self-report for phases 1–8. Stroke events were validated by Hospital Episode Statistics data linkage (discharge diagnosis or stroke-related procedure codes), General Practitioner’s confirmation or retrieval of hospital medical records up to end phase 8. Information from medical records was extracted onto standard forms for classifying suspected events according to protocol.^31^

Cancer incidence data for 1971 to March 2015 were obtained from the NHS Central Register for nearly all participants (n=10 297). Cancer sites in cancer registry data were coded according to ICD-9 in 1971–1994 and ICD-10 from 1995 onwards. Incidence of malignant cancer was defined as ICD-9 codes 140–208 or ICD-10 codes COO-C97.

Based on the above information, we determine between phases 1–9 whether respondents have yet been diagnosed with any chronic disease. Chronic disease is represented as a time-varying indicator, that is, after diagnosis of chronic disease at any given phase, respondents are identified as chronically ill at all subsequent phases.

### Psychosocial working conditions

Participants self-reported job demands, decision authority, skill discretion and social support at baseline (phase 1) using Karasek’s Job Content Questionnaire.^32^ Psychological job demands were operationalised by four items such as ‘Do you have to work very fast?’ (Cronbach’s α=0.67). Skill discretion and decision authority are regarded as being indicative of job control (Cronbach’s α=0.84). Skill discretion is measured by six items such as ‘Do you have to do the same thing over and over again’ and decision authority by nine items asking among others ‘Do you have a choice in deciding how to do your work?’. Social support at work consists of six items combining aspects of support from colleagues and superiors (Cronbach’s α=0.79). For each item, respondents rated whether it is ‘often’, ‘sometimes’, ‘seldom’ or ‘never/almost never’ the case. The final scales for job demands, skill discretion, decision authority and social support were converted to range from 0 to 100, with higher values indicating higher job demands, skill discretion and so on. For each of the four scales, continuous scores were divided into tertiles.

Participants grouped in the highest tertile of skill discretion, decision authority or work social support are categorised as having favourable working conditions, as does the lowest tertile of job demands. We observe whether respondents were exposed to favourable working (yes/no) at baseline for each of the four indicators of psychosocial working conditions.

### Covariates

Our covariates were time-constant and referred to the study baseline (phase 1). At baseline, all participants are working in the civil service at three *occupational grade levels*: ‘Administrative’ (highest grade), ‘Professional/Executive’ (middle grade) or ‘Clerical/Support’ (lowest grade). We included respondents’ *gender* and *educational level*. Educational level was measured in three categories, ‘low’ (‘General Certificate of Education (GCE) O-level or lower’; lower secondary education or lower), ‘middle’ (‘GCE A-level or equivalent’; upper secondary education) and ‘high’ (‘Degree level’; tertiary education). If educational level at baseline was unknown, we used information from phase 5 if available. *Work status of the partner* differentiated the following four categories: no partner; partner employed; partner unemployed, housework or no work; partner with missing information on employment status. Mental health was measured by the GHQ30-depression subscale.^33^ Higher values indicate worse mental health. If baseline information was missing on mental health, we used information from phase 2 or 3 if available. Last, we include information whether someone was *still working in the civil service* in the last available phase before work exit. Participants moving to employment outside the civil service might encounter different pension regulations. For example, state pension age was 60 years for men in the civil service but 65 years for men outside the civil service.

### Statistical analyses

The data allowed observing participants for approximately 20 years. The mean follow-up time was 14.1 years and we analyse 127 444 person-years. We observe three types of work exit: retirement, health-related exit and unemployment. ‘Other exit’ and ‘death’ are included as competing exit routes. This implies, when analysing, for example, the event of retirement, all other exit routes, that is, health-related exit, unemployment, other exit and death, are treated as competing events.

Several approaches to modelling competing risks are discussed in the literature. The cause-specific proportional hazard model and the subdistribution proportional hazards model are two of the best known and most widely used.^11 34^ Their assumptions, differences and similarities are discussed in detail elsewhere.^34 35^ We present results from the cause-specific Cox proportional hazard model (*stcox* in Stata). With this approach, competing events are censored, whereas they are kept in the at-risk population when modelled with the subdistribution proportional hazards model.^34^ For comparison, the results using a subdistributional hazard function, estimated with the Fine and Gray method (*stcrreg* in Stata),^36^ can be found in the online supplementary material table 2. The cause-specific Cox model assumes proportionality of the hazard. We relax this assumption by including chronic disease as a time-varying variable.^35^ For some working conditions, the proportionality assumption was not met in the models analysing exit through retirement, but were met for health-related exit and unemployment. The log-log plots of survival indicated that non-proportionality was restricted to young age especially. We decided to continue estimating Cox models because the non-proportionality did not affect our results; the results presented here were very comparable to those using the Weibull distribution (see online supplementary table 3).

10.1136/oemed-2017-104452.supp1 Supplementary file 1

Type of work exit was self-reported in this study, meaning that respondents’ subjective experiences might diverge from objective exit types. Therefore, we additionally analyse all types of exits combined (category ‘any exit from work’). Our indicator of chronic disease was time-varying. Thus, as it cannot be assumed that observations within each subject are independent, we specified robust SEs for all models.^35^

If information on the covariates was missing, which was the case for 6% of the observations, we imputed this information using imputation for chained equations (*mi impute chained* in Stata). We generated 20 imputed datasets, using the prediction model of work exit. The average relative variance increase (RVI) due to imputation is low (average RVI=0.01). All results presented here refer to the imputed datasets.

All analyses were adjusted for the covariates described above. We report χ^2^ test statistics to assess whether working conditions significantly moderated the relation between chronic disease and work exit (see table 3). Interactions would be regarded significant if the reported χ^2^ statistic (1 degree of freedom) was larger than the critical value of 3.841.

## Results

From all participants included in our study, 5629 left work during follow-up (62%). Retirement was the most frequent transition out of work (n=4254), followed by health-related exit (n=618) and unemployment (n=254). We censored respondents who had another exit from work (n=296) or died during follow-up (n=207).

The prevalence of chronic disease was less than 2% in phase 2 and increased to nearly 30% by phase 9 (see table 1a). Regarding the four chronic diseases separately, we see that heart disease and diabetes increase with about the same extent over time. Cancer is less frequent and very few people experience a non-fatal stroke.

**Table 1a:** Prevalence of chronic disease, phases 1–9 (n=9040)

	Chronic disease	Heart disease	Diabetes	Cancer	Stroke
Phase 1	0%	0%	0%	0%	0%
Phase 2	1.8%	1.1%	0.3%	0.4%	0.0%
Phase 3	5.3%	2.1%	2.3%	1.0%	0.1%
Phase 4	8.5%	3.8%	3.1%	1.9%	0.3%
Phase 5	12.9%	5.3%	5.1%	3.1%	0.5%
Phase 6	15.8%	6.5%	5.4%	4.6%	0.8%
Phase 7	21.1%	7.9%	8.2%	6.6%	1.2%
Phase 8	25.5%	9.6%	9.5%	9.0%	1.6%
Phase 9	29.9%	11.4%	13.4%	9.0%	1.6%

Chronic disease indicates whether participants have one or more of the four chronic diseases.

Table 1b shows the descriptive characteristics of our sample. We report the number of complete cases for each variable, incomplete cases were imputed. Forty-one per cent of our sample had low job demands. About 52% had high decision authority and 49% had high skill discretion. Fifty-three per cent had high work social support. About one-third of the participants were women and about half of the participants worked at a middle occupational grade level (Professional/Executive). Two-thirds our sample was still working in the civil service before work exit. Our sample comprises slightly more participants with high educational level. Most participants had a partner who was in employment and approximately one quarter had no partner. The mean depression score in our sample was 1.14.

**Table 1b:** Descriptive statistics of sample

	Percentage (N)	Mean (SD)
Work exit (n=9040)		
No	37.7 (3411)	
Yes	62.3 (5629)	
Route of work exit		
Retirement	75.6 (4254)	
Health-related exit	11.0 (618)	
Unemployment	4.5 (254)	
Other	5.2 (296)	
Death	3.7 (207)	
Age at work exit		
Retirement		59.8 (4.3)
Health-related exit		56.1 (5.7)
Unemployment		56.1 (5.1)
Low job demands (n=9035)		
No	59.0 (5331)	
Yes	41.0 (3704)	
High decision authority (n=9035)		
No	48.2 (4352)	
Yes	51.8 (4683)	
High skill discretion (n=9037)		
No	51.3 (4638)	
Yes	48.7 (4399)	
High work social support (n=9031)		
No	46.9 (4238)	
Yes	53.1 (4793)	
Gender (n=9040)		
Men	68.3 (6172)	
Women	31.7 (2868)	
Occupational grade level (n=9040)		
Administrative (high)	30.6 (2769)	
Professional/Executive (middle)	48.7 (4404)	
Clerical/Support (low)	20.7 (1867)	
Still in civil service (n=9040)		
No	34.0 (3070)	
Yes	66.0 (5970)	
Educational level (n=8382)		
Low	34.5 (2889)	
Middle	24.8 (2075)	
High	40.8 (3418)	
Spouse employment status (n=9012)		
No partner	25.0 (2257)	
Partner employed	42.8 (3858)	
Partner unemployed, housework or no work	13.2 (1187)	
Partner missing employment status	19.0 (1710)	
Depression score (GHQ score, n=9040)		1.14 (1.85)

Scores on variables with n<9040 were imputed.

GHQ, General Health Questionnaire.

We report the associations for the covariates in the fully adjusted model in the online supplementary table 1. Women, compared with men, had an increased risk to exit work through retirement and health-related exit (latter not significant), but had reduced risks of unemployment. Those with middle or low occupational grade level, in contrast to high occupational grade, had significantly reduced risks of retirement. The risk of health-related exit and unemployment for these two groups was increased, but not all differences with the high occupational level were significant. Those who were still working in the civil service, rather than having left it, had increased risks of all forms of work exit. Those with middle, compared with low, educational level had higher risks of retirement, whereas those with high, compared with low, educational level had reduced risks of retirement. Those with a partner generally had reduced risks of any form of work exit compared with those without a partner. Higher scores on the depression scale were related with increased risks of health-related exit.

Table 2 shows the fully adjusted models for any exit from work (all types combined) as well as retirement, health-related exit and unemployment separately. Being diagnosed with chronic disease is associated with significantly increased risks of work exit (HR 1.27, 95% CI 1.18 to 1.36). Chronic disease especially increases the risk of health-related exit (HR 2.42, 95% CI 2.01 to 2.92). It does not significantly relate to the risk of retirement or unemployment.

**Table 2:** Cause-specific Cox models for routes of work exit

	Any exit from work (5629/9040)	Retirement (4254/9040)	Health-related exit (618/9040)	Unemployment (254/9040)
	HR (95% CI)	HR (95% CI)	HR (95% CI)	HR (95% CI)
Chronic disease				
No	1.0	1.0	1.0	1.0
Yes	**1.27 (1.18 to 1.36)**	0.98 (0.91 to 1.07)	**2.42 (2.01 to 2.92)**	1.08 (0.75 to 1.53)
Low job demands				
No	1.0	1.0	1.0	1.0
Yes	0.97 (0.91 to 1.02)	0.99 (0.93 to 1.07)	0.90 (0.75 to 1.08)	0.84 (0.64 to 1.10)
High decision authority				
No	1.0	1.0	1.0	1.0
Yes	0.95 (0.89 to 1.00)	0.98 (0.92 to 1.05)	0.87 (0.72 to 1.04)	0.78 (0.59 to 1.03)
High skill discretion				
No	1.0	1.0	1.0	1.0
Yes	**0.90 (0.84 to 0.96)**	**0.91 (0.84 to 0.98)**	**0.68 (0.56 to 0.84)**	1.09 (0.80 to 1.47)
High work social support				
No	1.0	1.0	1.0	1.0
Yes	0.99 (0.94 to 1.04)	1.05 (0.98 to 1.12)	**0.79 (0.67 to 0.93)**	**0.71 (0.55 to 0.91)**

HRs with 95% CIs based on robust SEs. Observations, n=45 926. Average relative variance increase=0.01. Models are adjusted for gender, occupational grade level, still in civil service, educational level, partner’s employment status, depressive symptoms.

Bold values: p<0.05.

Favourable psychosocial working conditions might directly reduce the risk of work exit. Table 2 shows that high skill discretion in midlife, an indicator of job control, is significantly related to a decreased risk of work exit in general (HR 0.90, 95% CI 0.84 to 0.96) as well as reduced risk of retirement (HR 0.91, 95% CI 0.84 to 0.98) and health-related exit (HR 0.68, 95% CI 0.56 to 0.84). Decision authority is not significantly related to work exit. High work social support in midlife is significantly associated with reduced risks of health-related exit (HR 0.79, 95% CI 0.67 to 0.93) and unemployment (HR 0.71, 95% CI 0.55 to 0.91). Our results indicate that high job demands are generally related to lower risks of all types of work exit, but none of these associations is statistically significant.

Furthermore, we investigated whether favourable psychosocial working conditions reduced the risk of work exit among those diagnosed with chronic disease (see table 3). We do not find evidence for this hypothesis, with p>0.05 for all interaction terms, based on χ^2^ test. Workers with chronic disease had increased risks of any type of work exit and health-related exit regardless of whether or not they had favourable working conditions. If risk of work exit was reduced for those with favourable working conditions, this illustrates the main effect of working conditions, reported for example for high skill discretion. These findings replicate the direct effects presented in Table 2, but did not support our hypothesis of moderation.

**Table 3:** Cause-specific Cox models for routes of work exit investigating whether favourable working conditions moderate the relation between chronic disease and risk of work exit

		Any exit from work (5629/9040)	Retirement (4254/9040)	Health-related exit (618/9040)	Unemployment (254/9040)
		HR (95% CI)	HR (95% CI)	HR (95% CI)	HR (95% CI)
Chronic disease	Low job demands	χ^2^=0.04	χ^2^=1.16	χ^2^=3.74	χ^2^=0.11
No	No	1.0	1.0	1.0	1.0
No	Yes	0.96 (0.90 to 1.02)	1.01 (0.94 to 1.08)	**0.82 (0.65 to 0.99)**	0.83 (0.59 to 1.07)
Yes	No	**1.26 (1.14 to 1.37)**	1.02 (0.91 to 1.13)	**2.03 (1.49 to 2.57)**	1.02 (0.54 to 1.51)
Yes	Yes	**1.23 (1.10 to 1.36)**	0.94 (0.82 to 1.06)	**2.37 (1.74 to 3.01)**	0.95 (0.46 to 1.45)
Chronic disease	High decision authority	χ^2^=0.07	χ^2^=0.28	χ^2^=0.22	χ^2^=0.00
No	No	1.0	1.0	1.0	1.0
No	Yes	0.94 (0.88 to 1.00)	0.97 (0.90 to 1.05)	0.85 (0.67 to 1.02)	0.78 (0.54 to 1.02)
Yes	No	**1.25 (1.13 to 1.37)**	0.96 (0.85 to 1.07)	**2.35 (1.80 to 2.89)**	1.08 (0.59 to 1.58)
Yes	Yes	**1.20 (1.08 to 1.32)**	0.98 (0.86 to 1.09)	**2.17 (1.54 to 2.81)**	0.83 (0.36 to 1.30)
Chronic disease	High skill discretion	χ^2^=0.00	χ^2^=0.47	χ^2^=0.26	χ^2^=0.79
No	No	1.0	1.0	1.0	1.0
No	Yes	**0.90 (0.84 to 0.96)**	**0.90 (0.83 to 0.97)**	**0.66 (0.51 to 0.81)**	1.14 (0.77 to 1.50)
Yes	No	**1.26 (1.15 to 1.38)**	0.96 (0.85 to 1.06)	**2.35 (1.83 to 2.87)**	1.23 (0.68 to 1.78)
Yes	Yes	**1.14 (1.01 to 1.26)**	0.91 (0.79 to 1.02)	**1.73 (1.17 to 2.28)**	1.01 (0.42 to 1.60)
Chronic disease	High work social support	χ^2^=1.91	χ^2^=0.90	χ^2^=0.26	χ^2^=0.39
No	No	1.0	1.0	1.0	1.0
No	Yes	0.97 (0.91 to 1.03)	1.03 (0.96 to 1.11)	**0.77 (0.62 to 0.91)**	**0.68 (0.50 to 0.87)**
Yes	No	**1.20 (1.08 to 1.33)**	0.94 (0.83 to 1.06)	**2.33 (1.75 to 2.90)**	0.98 (0.51 to 1.44)
Yes	Yes	**1.28 (1.16 to 1.40)**	1.05 (0.93 to 1.17)	**1.96 (1.44 to 2.48)**	0.83 (0.41 to 1.26)

HRs with 95% CIs based on robust SEs. Observations n=45 926. Average relative variance increase=0.01. χ² test (1 degree of freedom) used to assess whether moderation is significant (critical value (p<0.05)=3.841). Models adjusted for gender, occupational grade level, still in civil service, educational level, partner’s employment status, depressive symptoms. Interactions between working conditions and chronic disease included in separate models; other working conditions included as covariates.

Bold values: p<0.05.

## Discussion

Using cause-specific Cox models, we showed that chronic disease was generally associated with increased risk of work exit and especially with increased risk of health-related exit. We do not find evidence that favourable psychosocial working conditions in midlife attenuated this association. However, high skill discretion directly related to reduced risk of work exit generally and retirement and health-related exit specifically. High work social support was related to lower risks of health-related exit and unemployment.

Some results corroborated our hypothesis that psychosocial working conditions could reduce the risk of work exit. Job control, represented by high skill discretion in midlife, reduced the risk of any exit from work, retirement and health-related exit. Several prior studies have shown that indicators of job control relate to work exit. Individuals with higher, compared with lower, skill discretion had lower risks of work exit^6 37^ as had those with higher decision authority (or job control, autonomy).^7 9 14 20^ This is, however, not unanimously replicated, because other authors have reported insignificant associations between decision authority and work exit.^5 10 22 26 37^ In our analyses, high work social support in midlife reduced the risk of health-related exit and unemployment. In previous research, social support was related to later retirement intentions and behaviour,^19 37^ but again, several other studies could not replicate this finding.^6 20 21^ Finally, we did not find evidence that lower psychosocial job demands in midlife reduced the risk of any types of work exit; results are, however, pointing in the expected direction. Our finding might be explained by previous studies showing that psychosocial job demands did not relate to retirement behaviour, but to retirement intentions.^19 20^ All in all, many previous studies have reported insignificant associations between job demands and work exit^13 14 19 20^ and, instead, emphasised job control as the key determinant. The importance of job control, especially skill discretion, is replicated in our study. Additionally, our results are particularly interesting, because our indicators of psychosocial job demands are measured in midlife. This might suggest that favourable psychosocial working conditions have advantageous long-term effects for work exit. In this respect, our study complements previous work measuring psychosocial working conditions closer to retirement.

We set out to provide more insights into the effect of the onset of chronic disease for work exit using prospective panel data. We found that chronic disease increased the risk of any type of work exit and specifically health-related exit. Similar findings were reported in prior studies, where poor health related to disability pension but not to early retirement or unemployment.^9 11^ In their literature review, van Rijn and colleagues reported an increased risk of disability pension for those with poor health as well as those with chronic disease.^4^ With regard to early retirement or unemployment, however, their meta-analysis assessed that only few studies found significant associations between poor health or chronic disease and the risk of unemployment or early retirement.^4^ Other factors, such as financial arrangements, might be more important in explaining early retirement.^11^ Moreover, both good health and poor health might stimulate early retirement, but the mechanisms, respectively voluntary exit to enjoy leisure time and involuntary exit out of necessity, would be different.^38^

We did not find support for our expectation that favourable working conditions could moderate the negative effect chronic disease had for work exit. Few studies have previously examined this moderation. Boot and colleagues reported that physical job demands as well as psychosocial resources—that is, a combined scale of items regarding decision authority, skill discretion and social support—mitigated the relation between chronic disease and remaining in paid work.^13^ Moreover, research by Leijten *et al* revealed that only those with high autonomy (ie, here: decision authority), compared with those with low autonomy, had reduced risks of exiting through disability benefits^22^ or increased likelihood of sickness absence.^13 22 26^ No other working conditions were found to be moderators. The previous three articles measured the working conditions shortly before work exit or sickness absence, with intervals varying between 1 and 3 years, whereas we measured working conditions in midlife, on average 14 years prior to exit. This might imply that psychosocial working conditions attenuate negative effects of chronic disease in the short term, but not in the long term. Future research could investigate whether the possible moderating role of favourable working conditions for work exit among those with poor health is enduring.

Our research contributes to prior studies on the association between chronic disease, psychosocial working conditions and three specific types of work exit. Our results illustrate that associations with work exit are not universal, but might affect one type of exit and not the other. This underlines the importance of differentiating possible exit routes.

In our study, the exit routes are distinguished based on self-reports. Especially regarding health-related exit, it is important to consider that we cannot be certain that participants leaving work through this exit route are in receipt of disability/sickness-related benefits. In the UK, the age at which State Pension could be claimed was until recently 65 years for men and 60 years for women. Early retirement is possible through many occupational pension schemes. For our participants in the civil service, occupational pension age was 60 years for men and women, but could often be claimed early, including in cases of sickness or disability.^39^ Disability benefits in the UK are available as earnings replacement, but are, unlike in other European countries, not earnings related. Therefore, disability benefits are generally only attractive to individuals with low income or those who already received occupational pensions.^40^

There are some possible limitations of our study. The Whitehall II study relies on a sample of civil servants in London and is not representative of the general population. Psychosocial working conditions comprised extensive measures and were recorded in participants’ midlife, allowing us to assess long-term effects. Unfortunately, we did not have information on later-life working conditions.

Our measure of chronic disease comprises four illnesses (CHD, diabetes, stroke and cancer). It is advantageous that information on these diseases was validated through health measures, hospital or General Practitioner (GP) records. However, the restricted information on four diseases could reduce the comparability to other studies using a wider range of self-reported measures of longstanding illnesses.^13–15 22 26^ Moreover, several participants might suffer from a chronic disease besides the ones included in our measure, for example, musculoskeletal disease. This implies that the HRs for chronic disease we presented might be an underestimation of the true effect. Additional analyses (not shown) using a self-reported measure of longstanding illnesses supported this idea, but results for the hypothesised relationships did not substantially differ from the ones presented here.

All analyses were adjusted for covariates known to relate to work exit. We did not investigate whether associations differed according to, for example, gender and socioeconomic status. Our analyses can, therefore, be interpreted as overall effects for men and women from different socioeconomic status only. Investigating differences between groups was beyond the scope of this article and might be an interesting endeavour for future research.

To summarise, we showed that chronic disease increased the risk of work exit, specifically of health-related exit and that good psychosocial working conditions could reduce the risk of work exit. Our study benefits from a thorough analysis of various exit routes and emphasises the need to regard these separately in future research. Moreover, future researchers and policy advisors should put more focus on the group of chronically ill workers and their special requirements in the workplace in order to advance the discussion on delaying work exit and extended working lives.
