# Supplementary material for: Can favourable psychosocial working conditions in midlife moderate the risk of work exit for chronically ill workers? A 20-year follow-up of the Whitehall II study
Source: Occup Environ Med. 2017 Oct 17;75(3):183–90. doi: 10.1136/oemed-2017-104452 (PMC5869452; doi:10.1136/oemed-2017-104452)
Supplement: Supplementary file 1 [file oemed-2017-104452supp001.pdf]

Supplementary Tables

Supplementary Table 1: Cause-specific Cox models for routes of work exit. Hazard ratios with 95% confidence intervals based on robust standard errors. Observations n=45,926. Average relative variance increase (RVI)=0.01.

	Any exit from work (5,629/9,040) HR [95% CI]	Retirement (4,254/9,040) HR [95% CI]	Health-related exit (618/9,040) HR [95% CI]	Unemployment (254/9,040) HR [95% CI]
**Gender**				
Men	1.0	1.0	1.0	1.0
Women	**1.17 [1.10,1.26]**	**1.17 [1.08,1.27]**	1.15 [0.95,1.40]	**0.66 [0.48,0.92]**
**Occupational grade level**				
Administrative (high)	1.0	1.0	1.0	1.0
Professional/Executive (middle)	**0.91 [0.84,0.97]**	**0.84 [0.77,0.91]**	1.22 [0.94,1.57]	**1.54 [1.09,2.18]**
Clerical/Support (low)	**0.52 [0.47,0.58]**	**0.42 [0.37,0.48]**	1.26 [0.89,1.79]	1.60 [0.97,2.64]
**Still in civil service**				
No	1.0	1.0	1.0	1.0
Yes	5.01 [4.64,5.41]	**5.80 [5.30,6.35]**	**5.20 [4.00,6.76]**	**2.39 [1.72,3.33]**
**Educational level**				
Low	1.0	1.0	1.0	1.0
Medium	**1.12 [1.04,1.20]**	**1.14 [1.05,1.24]**	0.89 [0.72,1.11]	0.97 [0.69,1.36]
High	**0.92 [0.85,0.98]**	**0.88 [0.81,0.96]**	0.86 [0.69,1.06]	0.93 [0.67,1.29]
**Spouse employment status**				
No partner	1.0	1.0	1.0	1.0
Partner employed	**0.85 [0.80,0.91]**	**0.90 [0.83,0.98]**	**0.72 [0.59,0.86]**	**0.66 [0.48,0.89]**
Partner unemployed, housework or no work	**0.75 [0.68,0.83]**	**0.80 [0.71,0.90]**	0.78 [0.59,1.04]	**0.37 [0.22,0.63]**
Partner missing employment status	**0.87 [0.80,0.94]**	0.91 [0.83,1.00]	**0.66 [0.51,0.86]**	0.75 [0.52,1.08]
**Depression score** (GHQ score)	1.01 [1.00,1.03]	0.99 [0.98,1.01]	**1.11 [1.08,1.15]**	1.04 [0.98,1.10]

Note: Results of covariates reported only. Direct effect of chronic disease and psychosocial working conditions included in model, reported in Table 2.

Bold values: $p<.05$

Supplementary Table 2: Subdistributional hazard models using the Fine & Gray method to estimate risk of work exit through different exit routes. Subhazard ratios with 95% confidence intervals. Observations n=45,926. Results based on 10 imputed datasets, largest average relative variance increase (RVI)=0.016.

	Retirement (4,254/9,040) SHR [95% CI]	Health-related exit (618/9,040) SHR [95% CI]	Unemployment (254/9,040) SHR [95% CI]
**Chronic disease**			
No	1.0	1.0	1.0
Yes	**0.90 [0.82,0.98]**	**2.57 [2.13,3.09]**	1.09 [0.77,1.54]
**Low job demands**			
No	1.0	1.0	1.0
Yes	1.05 [0.98,1.12]	0.94 [0.78,1.13]	0.87 [0.66,1.14]
**High decision authority**			
No	1.0	1.0	1.0
Yes	1.07 [1.00,1.15]	0.89 [0.75,1.07]	0.80 [0.61,1.07]
**High skill discretion**			
No	1.0	1.0	1.0
Yes	0.96 [0.89,1.04]	**0.70 [0.57,0.86]**	1.13 [0.84,1.53]
**High work social support**			
No	1.0	1.0	1.0
Yes	**1.10 [1.03,1.17]**	**0.79 [0.67,0.93]**	**0.71 [0.55,0.92]**

Note: Models are adjusted for gender, occupational grade level, still in civil service, educational level, partner's employment status, depressive symptoms.

Bold values: p<.05

Supplementary Table 3: Cause-specific parametric mixture model using the Weibull distribution to estimate risk of work exit through different exit routes. Hazard ratios with 95% confidence intervals based on robust standard errors. Observations n=45,926. Average relative variance increase (RVI)=0.01.

	Any exit from work (5,629/9,040) HR [95% CI]	Retirement (4,254/9,040) HR [95% CI]	Health-related exit (618/9,040) HR [95% CI]	Unemployment (254/9,040) HR [95% CI]
**Chronic disease**				
No	1.0	1.0	1.0	1.0
Yes	**1.24 [1.15,1.33]**	0.95 [0.86,1.04]	**2.42 [2.00,2.91]**	1.02 [0.71,1.45]
**Low job demands**				
No	1.0	1.0	1.0	1.0
Yes	0.96 [0.91,1.02]	0.99 [0.92,1.07]	0.90 [0.75,1.08]	0.84 [0.64,1.10]
**High decision authority**				
No	1.0	1.0	1.0	1.0
Yes	0.94 [0.89,1.00]	0.97 [0.90,1.05]	0.87 [0.73,1.05]	0.78 [0.59,1.03]
**High skill discretion**				
No	1.0	1.0	1.0	1.0
Yes	**0.89 [0.84,0.96]**	**0.90 [0.83,0.98]**	**0.68 [0.56,0.83]**	1.08 [0.79,1.46]
**High work social support**				
No	1.0	1.0	1.0	1.0
Yes	0.99 [0.94,1.05]	1.05 [0.98,1.13]	**0.79 [0.67,0.93]**	**0.71 [0.55,0.91]**
Shape parameter (log(p))	2.63 [2.60, 2.66]	2.80 [2.76,2.83]	2.06 [1.95,2.17]	2.08 [1.94,2.22]

Note: Models are adjusted for gender, occupational grade level, still in civil service, educational level, partner's employment status, depressive symptoms.

Bold values: p<.05

Supplementary Table 4: Work exit through unemployment and other exit. Hazard ratios with 95% confidence intervals based on robust standard errors. Observations n=45,926. Average relative variance increase (RVI)=0.01.

	Unemployment (254/9,040) HR [95% CI]	Other exit (296/9,040) HR [95% CI]
**Chronic disease**		
No	1.0	1.0
Yes	1.08 [0.75,1.53]	1.03 [0.73,1.45]
**Low job demands**		
No	1.0	1.0
Yes	0.84 [0.64,1.10]	0.84 [0.65,1.09]
**High decision authority**		
No	1.0	1.0
Yes	0.78 [0.59,1.03]	**0.77 [0.60,0.99]**
**High skill discretion**		
No	1.0	1.0
Yes	1.09 [0.80,1.47]	0.77 [0.59,1.00]
**High work social support**		
No	1.0	1.0
Yes	**0.71 [0.55,0.91]**	0.86 [0.68,1.08]
**Gender**		
Men	1.0	1.0
Women	**0.66 [0.48,0.92]**	**2.52 [1.91,3.32]**
**Occupational grade level**		
Administrative (high)	1.0	1.0
Professional/Executive (middle)	**1.54 [1.09,2.18]**	0.95 [0.71,1.28]
Clerical/Support (low)	1.60 [0.97,2.64]	**0.27 [0.16,0.45]**
**Still in civil service**		
No	1.0	1.0
Yes	**2.39 [1.72,3.33]**	**1.58 [1.19,2.11]**
**Educational level**		
Low	1.0	1.0
Medium	0.97 [0.69,1.36]	1.18 [0.84,1.65]
High	0.93 [0.67,1.29]	1.30 [0.95,1.79]
**Spouse employment status**		
No partner	1.0	1.0
Partner employed	**0.66 [0.48,0.89]**	0.90 [0.66,1.22]
Partner unemployed, housework or no work	**0.37 [0.22,0.63]**	0.74 [0.47,1.17]
Partner missing employment status	0.75 [0.52,1.08]	1.04 [0.73,1.48]
**Depression score** (GHQ score)	1.04 [0.98,1.10]	0.96 [0.89,1.02]

Note: Bold values: $p<.05$
